# Behavior of Prions in the Environment: Implications for Prion Biology

**DOI:** 10.1371/journal.ppat.1003113

**Published:** 2013-02-07

**Authors:** Shannon L. Bartelt-Hunt, Jason C. Bartz

**Affiliations:** 1 Department of Civil Engineering, University of Nebraska-Lincoln, Peter Kiewit Institute, Omaha, Nebraska, United States of America; 2 Department of Medical Microbiology and Immunology, Creighton University, Omaha, Nebraska, United States of America; Washington University School of Medicine, United States of America

## Emergence of Prion Diseases

Prion diseases are infectious, potentially zoonotic neurodegenerative diseases of animals including humans that are inevitably fatal and are caused by prions. Prions are comprised of a misfolded isoform of the normal prion protein, PrP^C^, into the infectious conformation, PrP^Sc^ [1]. Of the known prion diseases, chronic wasting disease (CWD) of deer, elk, and moose is emerging. CWD was first identified in captive deer in the front range of Colorado and Wyoming in the 1960s and has since been identified in captive and free-ranging cervids in 20 states, two Canadian provinces, and South Korea (for latest disease distribution please see http://www.nwhc.usgs.gov/disease_information/chronic_wasting_disease/index.jsp). While there is evidence of the spread of CWD along known cervid home ranges, the mechanism underlying the emergence of CWD in geographically isolated areas is not understood. The prevalence of CWD within an affected population is generally lower than 5%; however, there are reports of incidence rates that approach 50%. Transmission of CWD can occur horizontally or through CWD-contaminated environments, but the relative contribution of each mode in the overall transmission of CWD is unknown [2]. Since effective control measures are not available, it is likely that CWD will continue to spread in North America. The effect of this on the well-being of the cervid population and the risk of transmission to other species is not known.

## Prions Are Released into the Environment and Remain Infectious

It has long been observed that indirect lateral transmission of scrapie can occur, and recent evidence also demonstrates indirect lateral transmission of CWD [3]. One factor influencing the environmental transmission of prion diseases is the long-term survival of prions in the environment. Epidemiological studies indicate numerous instances of scrapie recurrence upon reintroduction of animals on farms previously exposed to scrapie. Scrapie recurrence was documented following fallow periods of 1–16 years [4], and pastures can retain infectious CWD prions at least 2 years after exposure [5].

Prions are shed from diseased hosts in a diverse set of biologic matrices, including feces, urine, saliva, blood, skin, milk, placenta, and nasal mucus. A comprehensive review of prion shedding was conducted by Gough and Maddison [6]. Prion shedding can occur many months prior to clinical manifestation of the disease [7]. Prions also enter the environment after decomposition of diseased animal carcasses [5]. The disposal of diseased cattle during bovine spongiform encephalopathy (BSE) outbreaks, both in the past and in potential future disposal events, serves as another environmental source of prions. Uptake of prions to naïve hosts can occur via ingestion or inhalation of contaminated material, although the routes of natural exposure remain uncertain [8].

Recently, scrapie and CWD prions have been detected in environmental samples by protein misfolding cyclic amplification (PMCA). One of two water samples collected from a CWD-endemic area in Colorado was determined to be positive for CWD [9]. Maddison et al. [10] detected scrapie prions on swabs collected from metal, plastic, and wooden surfaces on a scrapie-endemic farm. In the Maddison et al. [10] study, it is not clear whether the scrapie prions associated with the surfaces were co-transported via soil or dust. To our knowledge, no study has investigated the occurrence of CWD or scrapie prions in soil samples collected from areas with known incidence of prion disease.

## In the Environment, Prions Can Bind to Soil

Prions shed into the environment will interact with soil. Given the close contact that animals, especially ruminants, have with soil through routine behaviors, including ingestion of soil via feeding and mineral supplementation, there is significant opportunity for transmission of prions via soil. Prions appear to have an affinity for quartz sands and soils and a particularly strong affinity for clay minerals [11]. The biological matrix that prions enter the environment (e.g., urine versus animal carcass) influences the kinetics of prion sorption to soil. Prions sorb to soil more slowly in complex biological matrices compared to prions in simple matrices, likely due to competitive interactions [12]. In addition, the kinetics of PrP^Sc^ binding to soil can be influenced by the prion strain [13]. Sorbed prions are resistant to desorption via detergent and chaotropic treatments. As with other proteins, prion sorption is most likely a combination of electrostatic attraction and hydrophobic interactions. Studies using recombinant prions have identified electrostatic attraction between positively charged peptides and negatively charged mineral surfaces as the most significant adsorption mechanisms [14]. Because the N-terminal domain of the prion protein is known to be flexibly disordered and contains a high number of positively charged amino acids, it may play a significant role in electrostatic attraction to negatively charged mineral surfaces. The N-terminal domain is lost upon desorption of PrP^Sc^ from clay, but it is not needed for prion adsorption or infectivity [11]. Recombinant PrP has a high affinity for organic matter, equal to or greater than that calculated for mineral surfaces [11].

The three-dimensional structure of PrP^Sc^ remains unknown; therefore, it is a challenge to model the specific mechanisms that are significant in PrP^Sc^ adsorption to soil. PrP^Sc^ is aggregated, and changes in the aggregation state could occur with soil binding, potentially affecting infectivity. One study did find that recPrP does not form β-sheets or self-aggregate when adsorbed to clay [14]. More must be done to determine what conformational changes occur to PrP^Sc^ when it binds to soil or minerals and how these changes affect agent survival and infectivity.

## The Biologic Properties of Prions Can Be Altered by Attachment to Soil

The biologic properties of the prion protein, including conversion activity and infectivity, can be influenced by attachment to soil particles. Adsorption of CWD PrP^Sc^ to soil reduces prion conversion activity via PMCA [15]. The observed decrease in the ability of prions to convert upon binding to certain soils could be due to a number of factors, including conformational changes in PrP^Sc^ structure, interference with PrP^C^/PrP^Sc^ interactions, or a change in PrP^Sc^ stability that may occur upon binding to soil.

Several studies have investigated the role of soil on prion infectivity. Johnson et al. [16] investigated the infectivity of the hyper strain of transmissible milk encephalopathy (HY TME) bound to montmorillonite (Mte) clay particles via intracerebral inoculation. Bioassay results demonstrated a 10-day decrease in incubation period for PrP^Sc^-Mte complexes when compared to PrP^Sc^ inocula without Mte. A second study investigating infectivity of PrP^Sc^ bound to Mte via oral routes also demonstrated an increase in infectivity relative to clay-free controls [17]. Saunders et al. [15] conducted bioassay experiments using HY TME PrP^Sc^ bound to a silty clay loam soil and demonstrated a 14-day extension in incubation period and a 1.3 log reduction in titer, as determined by end point dilution, for soil-bound HY TME prions. This data is consistent with the calculated decrease in PMCA conversion efficiency for soil-bound HY TME PrP^Sc^. The discrepancies between observed differences in soil-bound prion infectivity may be explained by differences in experimental design, such as preparation of PrP^Sc^ inocula. Most importantly, all of these studies consistently demonstrate that prions sorbed to soil remain highly infectious and that binding to soil can alter prion infectivity.

## The Impact of the Environment on Prion Disease Transmission

**Figure 1:**
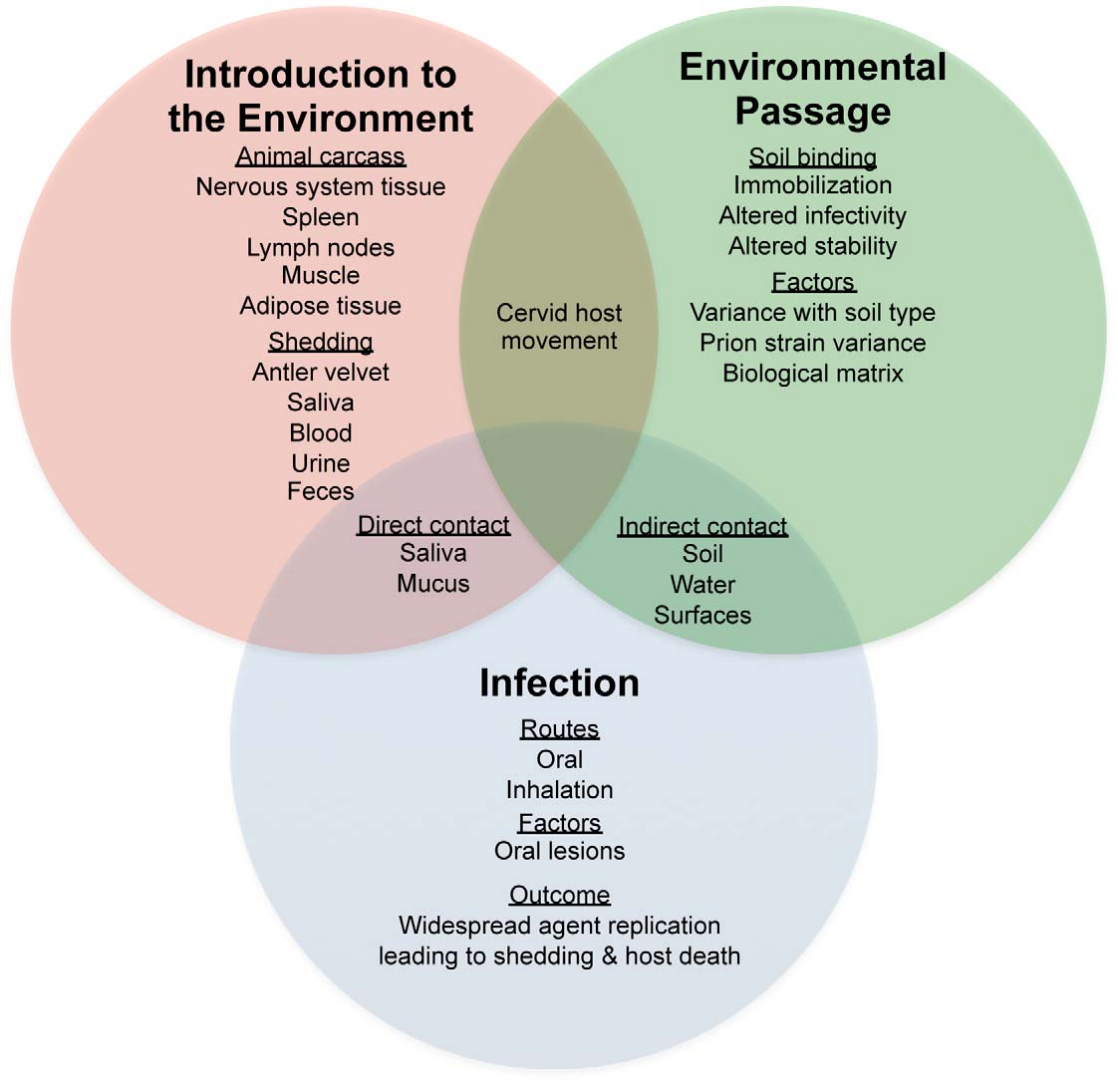
Factors influencing horizontal transmission of prion disease in the environment.

The basic parameters of prion environmental interactions are only beginning to be described, and the effect of these interactions on prion transmission and pathogenesis are poorly understood. As shown in Figure 1, the interaction of prions in the environment is complex and must include consideration of the route of introduction for prions to the environment as well as the effects of soil properties and prion strain on prion interaction with soil. For example, the matrix of prion entry into the environment can influence the kinetics of prion binding to soil. Once bound to soil, prions do not readily disassociate from the soil particle and remain highly infectious. The implications of these important observations are that prions immobilized to soil may persist at the surface where transmission to a naïve host would be more likely to occur. Consistent with these observations, an increased incidence of CWD corresponds with geographic regions with soil types that have a high affinity to bind prions [18]. There is strong evidence for the existence of multiple strains of scrapie, and recent studies suggest that more than one strain of CWD exists [19]. Strain-specific interactions with the environment may result in preferential selection of strains that have properties that favor environmental persistence and transmission.
